# Open-path dual-comb spectroscopy of methane and VOC emissions from an unconventional oil well development in Northern Colorado

**DOI:** 10.3389/fchem.2023.1202255

**Published:** 2023-06-02

**Authors:** Griffin J. Mead, Eleanor M. Waxman, Daniel Bon, Daniel I. Herman, Esther Baumann, Fabrizio R. Giorgetta, Jacob T. Friedlein, Gabriel Ycas, Nathan R. Newbury, Ian Coddington, Kevin C. Cossel

**Affiliations:** ^1^ National Institute of Standards and Technology, Spectrum Technology and Research Division, Boulder, CO, United States; ^2^ Colorado Department of Public Health and Environment, Denver, CO, United States; ^3^ Department of Physics, University of Colorado, Boulder, CO, United States

**Keywords:** dual-comb spectroscopy, methane emissions, unconventional well development, open-path spectroscopy, Colorado Front Range, mid-infrared (IR) absorption

## Abstract

We present results from a field study monitoring methane and volatile organic compound emissions near an unconventional oil well development in Northern Colorado from September 2019 to May 2020 using a mid-infrared dual-comb spectrometer. This instrument allowed quantification of methane, ethane, and propane in a single measurement with high time resolution and integrated path sampling. Using ethane and propane as tracer gases for methane from oil and gas activity, we observed emissions during the drilling, hydraulic fracturing, millout, and flowback phases of well development. Large emissions were seen in drilling and millout phases and emissions decreased to background levels during the flowback phase. Ethane/methane and propane/methane ratios varied widely throughout the observations.

## 1 Introduction

Oil and natural gas extraction, processing, and consumption are a substantial source of global anthropogenic methane and volatile organic compound (VOC) emissions. In the past 2 decades within the United States, significant developments in extraction processes—in particular, horizontal drilling—have led to a several fold increase in oil and natural gas production volumes. Frequently, as exemplified with the oil and gas development of the Denver-Julesburg (DJ) basin in the Northern Colorado Front Range Urban Corridor, this results in resource extraction near businesses and homes. In addition to the climate impact of methane emissions, leaks from wells can pose health risks not only from explosions and fires, but also from the impacts of co-emitted VOCs (Field et al., 2014; Macey et al., 2014; McDuffie et al., 2016; McMullin et al., 2018; Garcia-Gonzales et al., 2019; Hecobian et al., 2019; Helmig, 2020). Some VOCs, such as benzene, are direct health hazards. Many others, including ethane and propane, contribute to increased ozone, formaldehyde, acetaldehyde, and secondary organic aerosol production, especially in urban areas such as the Front Range Urban Corridor where NO_x_ (NO + NO_2_) mixing ratios can be quite high (Abeleira and Farmer, 2017; Pfister et al., 2019). The oil and gas (O&G) sector contributes >80% of the United States emissions of ethane and propane, but the emissions trends for these gases aren’t well known and remain challenging to model (Tzompa-Sosa et al., 2019). Well sites therefore represent an interesting node in the energy economy where public health and environmental concerns intrinsically overlap. However, while methane emissions from existing wells and production and processing facilities have been studied extensively, emissions of methane and VOCs during the process of well installation (for example, drilling and hydraulic fracturing) aren’t as well known (Allen et al., 2013; Allen, 2014; Mitchell et al., 2015). Hundreds of new wells are installed every year in the DJ basin, emphasizing the importance of characterizing emissions during well development.

Traditionally, VOCs are measured using point sampling techniques, often with low temporal resolution. The most common method utilizes gas chromatography (GC), either offline with canister sampling (Hecobian et al., 2019) or *in situ* using pre-concentration, e.g., with a sorbent tube and thermal desorption (Tanner et al., 2006; Gilman et al., 2013; Pollack et al., 2021). Hourly measurements are common for these systems. While these techniques can characterize many VOCs in a single collection time period, they require significant calibration and up-keep which makes long-term emissions measurements challenging. High time resolution instruments, such as proton-transfer reaction mass spectrometry (Koss et al., 2017), have been used for direct emissions measurements (Edie et al., 2020) but still require substantial calibration efforts. As with all point measurements, however, even a perfectly calibrated instrument’s ability to sample a particular source is dictated by wind direction.

In contrast, the spectroscopic measurements over long open-air paths discussed here improve the detection probability for emissions plumes from a site, while remaining inherently calibrated without user intervention and without using consumables like gas chromatography columns or carrier gases. The open path measurement also increases the probability of intercepting a greater percent of emissions from a facility, which can improve the determination of emissions rates. Near-IR open-path dual-comb spectroscopy (DCS) (Coddington et al., 2016; Cossel et al., 2021) has been shown to enable long-term observations of methane emissions from oil and gas facilities (Coburn et al., 2018; Alden et al., 2019, 2020). Here, by operating in the mid-infrared, we demonstrate the viability of long-term VOC monitoring using a fieldable mid-infrared DCS system (Ycas et al., 2018, 2020). Simultaneous measurements of CH_4_ and heavier hydrocarbons allows not only leak detection and quantification, but also apportionment of emissions from different stages of the natural gas production process (which removes VOCs like ethane and propane from methane), as well as separation of fossil sources from biogenic methane (which is free of heavier hydrocarbons). (Yacovitch et al., 2014). VOC quantification may also lead to improved understanding of air quality impacts from well installation. To this end, we deployed a mid-IR DCS to an active oil and gas development for several periods over the course of 8 months to measure how concentrations of methane and VOCs change throughout the different installation phases of an unconventional oil and gas well. Further, we model correlations between methane and two O&G VOCs (either ethane or propane) to identify to identify the origin of methane plumes (Mead et al., 2023). Finally, we present emissions flux estimates for a large methane plume observed during the drilling phase.

## 2 Materials and methods

The mid-IR DCS system is similar to the one used in prior publications (Ycas et al., 2018, 2020) and provides a wide spectral bandwidth in the C-H stretch region from 3 to 4 μm, which covers many characteristic infrared-active transitions of methane and VOCs such as ethane and propane. Briefly, the system starts with two mode-locked, 200 MHz, Er:fiber frequency combs in the near-IR (Sinclair et al., 2015) and uses difference frequency generation (DFG) to produce the mid-IR dual-comb light. To achieve this, the output from each Er:fiber comb is split into two branches and amplified. One branch is spectrally broadened to produce light at 1 μm, and the other at 1.5 μm. The light from these two branches is combined and focused through a chirped periodically poled lithium niobate (PPLN) crystal, wherein DFG produces 3–5 mW of light in the required 3–4 μm band. Each comb is stabilized by detecting and locking to the carrier-envelope offset frequency using a turn-key fiber *f*-2*f* interferometry (Truong et al., 2016). To ensure optical coherence, a narrow-band 1.56 µm continuous-wave laser is locked to one comb and the second comb is locked to the continuous-wave laser. The repetition rates of the combs are locked to an rf synthesizer for long-term stability. The result is a tooth-resolved, fully coherent, broadband mid-IR DCS system (Ycas et al., 2020) capable of coherently averaging the atmospheric transmission spectrum for arbitrarily long periods.

**FIGURE 1:**
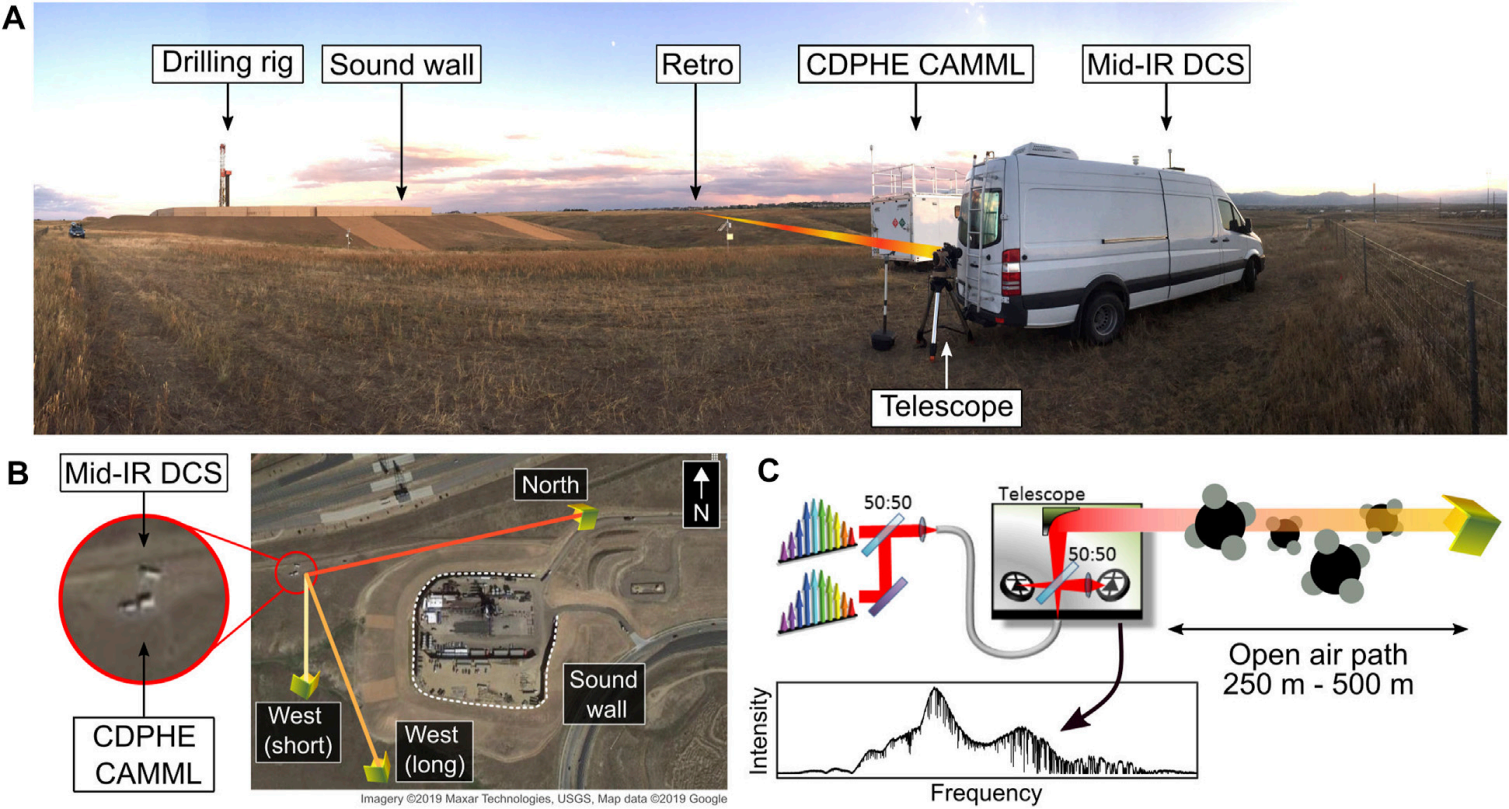
**(A)** A photograph of the measurement location, with the drilling rig, Colorado Department of Health and Environment (CDPHE) Air Monitoring Mobile Lab (CAMML), mid-IR system in the van, and illustrative beam paths. The gimbal mounted telescope is visible on the tripod by the rear door of the van. **(B)** Van and retroreflector locations relative to the well pad (Imagery ^©^2019 Maxar Technologies, USGS, map data ^©^2019 Google, accessed October 2019). **(C)** A diagram of the DCS open-path measurement technique. The two mid-infrared frequency combs are combined and projected with a telescope over an open-air path to a retroreflector. The return light is digitized with a photodiode. The path-averaged concentration of molecules along the path are calculated from the measured absorption spectra.

For open path measurements, the mid-IR light from each comb was combined and coupled into single-mode ZrF_4_ optical fiber and sent to a transmit-receive telescope that was custom-built in house (Cossel et al., 2021). In the telescope light was expanded from the fiber onto a collimating off-axis parabolic mirror (180-mm focal length) and transmitted over the air to a hollow corner cube retroreflector (retro). Light reflected from the retro was collected at the telescope and diverted with a 50:50 beam splitter to a thermo-electrically cooled HgCdTe photodetector which records the interference signal (interferogram) between the two mid-IR frequency combs. A diagram of the telescope and measurement path is shown in Figure 1C. Relying on the mutual coherence of the frequency combs, this interferogram down-converts the 20 THz optical spectrum to a 10 MHz band of rf spectra that can be easily digitized while still resolving each comb tooth. This signal is then phase corrected on a field-programmable gate array (FPGA) to remove any residual phase noise and then coadded for the measurement period. Scaling by the known repetition rates of the combs, the true optical spectrum can be determined from this averaged interferogram. The difference in comb repetition rates is 100 Hz, giving a native temporal resolution of 10 ms, though successive spectra are averaged for 1–5 min. The tooth spacing and coherence of the combs gives us a 0.0067 cm^−1^ spectral resolution, yielding 75,000 independent spectral elements across the atmospheric transmission spectrum with negligible instrument line shape.

In 2019 this DCS system was deployed to an unconventional well development site in Broomfield County, Colorado. An overview of the field deployment is shown in Figure 1A. The DCS laser system was mounted in a van with modest insulation and temperature control. The single-mode ZrF_4_ optical fiber conveyed the light from the DCS system to the gimbal-mounted transmit-receive telescope located outside the van. Several retroreflectors were located around the well site 250 m–500 m from the van, as shown in Figure 1B. Using the gimbal, the beam path was toggled between different retros, allowing for inspection of different borders around the site as the wind changed directions (data was collected along one beam path at a time).

**FIGURE 2:**
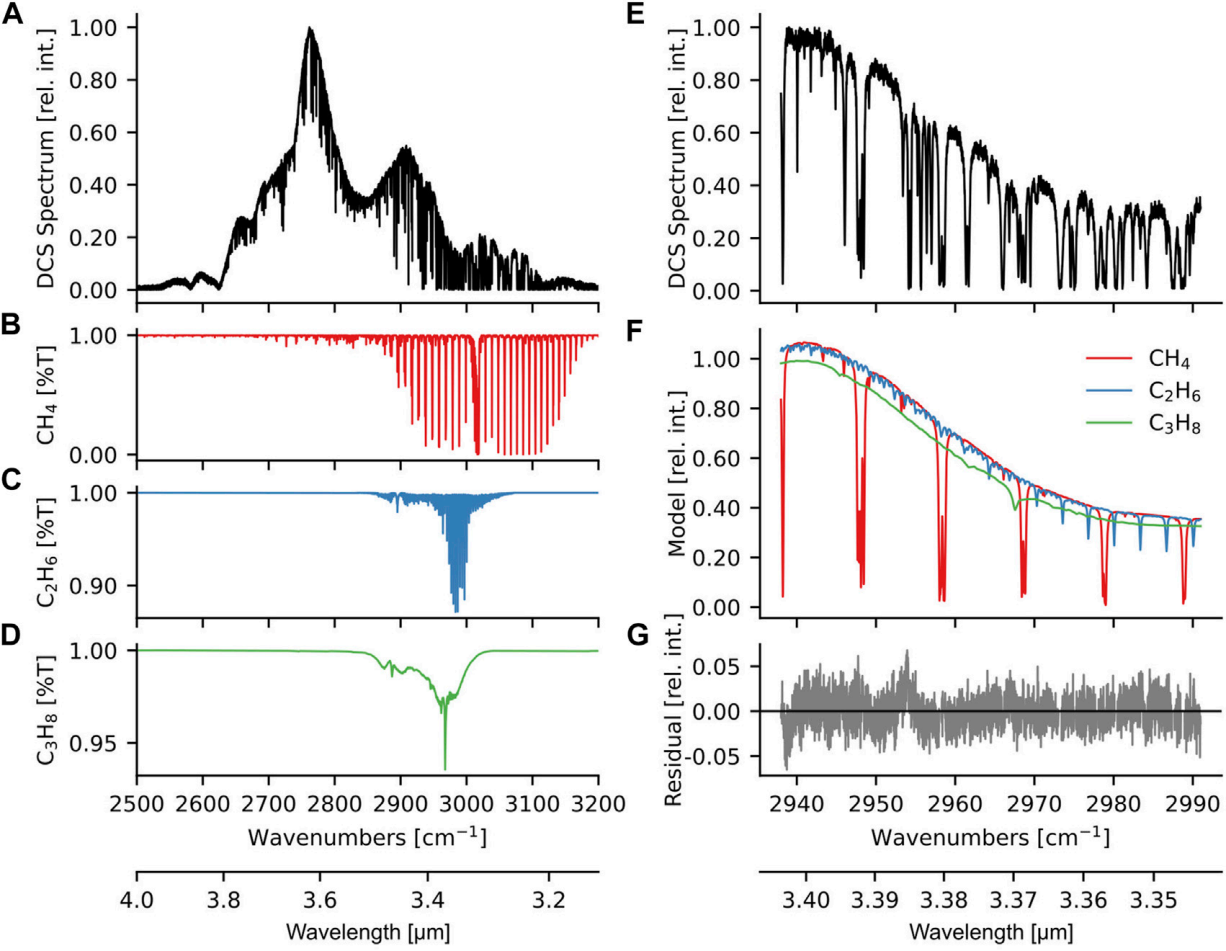
**(A)** A representative mid-IR spectrum recorded at the measurement location has a broad spectral bandwidth from 2,600 cm^-1^ to 3,100 cm^−1^. Downward pointing features are vibrational modes of different molecules in the beam path. **(B–D)** Transmission spectra of methane, ethane, and propane are plotted at the concentrations retrieved from the mid-IR spectrum in **(A)**. Features below 2,900 cm^−1^ are predominantly due to H_2_O and HDO. **(E)** A zoomed-in view of the fit region used for analysis in this work. **(F)** Modelled methane, ethane, and propane spectra fit to the data in **(E)**. Models are multiplied by a baseline fit; water is also fit for all data but not shown here. **(G)** Residuals for **(E)** after the models in **(F)** and water are subtracted.

A representative mid-IR spectrum measured at the site is shown in Figure 2A, with the constituent molecular spectra shown in Figures 2B–D. The broad spectral coverage, high spatial coherence, low intensity noise, inherent frequency calibration, and high spectral resolution afforded by the mode-locked laser frequency comb is currently difficult to match with other mid-infrared sources such as quantum and interband cascade lasers (QCLs/ICLs). This allows multi-species quantification in a single measurement which is key for tracer gas analysis of greenhouse gas emissions, as well as for monitoring dynamic processes like ozone production.

For each spectrum, data from 3.34 μm to 3.40 μm (Figure 2E) were fit with a fifth-order polynomial plus calculated spectra using the HITRAN 2020 (Gordon et al., 2021) and PNNL NWIR (Sharpe et al., 2004) spectral databases (Figure 2F) to obtain path-averaged concentrations of water, methane, ethane, and propane. The fit residuals are shown in Figure 2G. In addition to the fit species, other molecules are present in the data but not analyzed here. HDO and HCHO can be observed in the region closer to 3.6 μm (Herman et al., 2023). The C-H stretches of heavier alkanes such as butanes can be observed, especially in time periods with large methane enhancements, but spectral interference and the lack of distinct spectral features in these gasses made the retrieval sensitivity insufficient for most of the time periods. Different analysis methods could enable better detection of these species in the future (Cole et al., 2019). The precise path length between the telescope and retroreflector used for fitting the spectra was measured using a range finder with precision to better than 20 cm, and thus the path length measurement contributes negligible uncertainty to the final mixing ratio retrieval.

The DCS system was collocated with the Colorado Department of Health and Environment (CDPHE) Air Monitoring Mobile Lab (CAMML), as shown in Figure 1A, which provided valuable comparative data on methane and VOC mixing ratios. The CAMML measured VOC concentrations using a dual-column gas chromatography-flame ionization detector (GC-FID) instrument with a pre-concentrator system. The GC-FID collected for 45 min and analyzed hourly using a standard measurement protocol and daily calibration checks (Office of Air Quality Planning and Standards and Air Quality Assessment Division, 2019). Methane was measured with an off-axis integrated cavity output spectrometer (OA-ICOS) at 1 min time resolution. Meteorological data including wind speed and direction were measured at 1 min resolution with a compact weather station on roof of the CAMML system. All CAMML data is publicly available through CDPHE (Colorado Department of Public Health and Environment, 2020).

The unconventional well site consists of 18 boreholes that target the Niobrara shale rock formation at a depth of ∼2,450 m with a total length of ∼6,400 m. Horizontal drilling and hydraulic fracturing have become industry standards for unconventional oil and gas well development in Colorado (Zendehboudi and Bahadori, 2017). Horizontal drilling allows multiple individual wells to be drilled at the same location and to target a specific resource-rich strata, while hydraulic fracturing (fracking) increases the productivity of wells accessing low permeability (“tight”) formations. Generally, a new installation follows a series of phases. In the first phase (drilling), a drilling rig creates a vertical borehole to a predetermined depth, at which point lateral boreholes are horizontally drilled into the target formation. Throughout the drilling process the drill bit is periodically retracted from the well and the borehole is strengthened and isolated from the environment by installing metal casings and cement. Once the final bore length is drilled, a well head is installed; the fracking phase can then proceed either in stages (where a portion of the well is selectively plugged and fractured) or along the entire lateral borehole. Fracking fluid pressurizes the well to several MPa, generating cracks and fissures in the formation. The average well requires several million liters of fracking fluid; proppant (sand) carried by the fluid penetrates cracks, forcing them to remain open. Because the wells at this site were plugged and fractured in stages, a third phase (millout) is required to remove the fracking plugs after fracking is complete. Finally, pressure in the well is reduced and the injected fluid is forced back to the surface by the internal pressure of the borehole (flowback), after which the well begins production of oil and/or gas.

## 3 Results

### 3.1 Time series and comparison to CAMML

**FIGURE 3:**
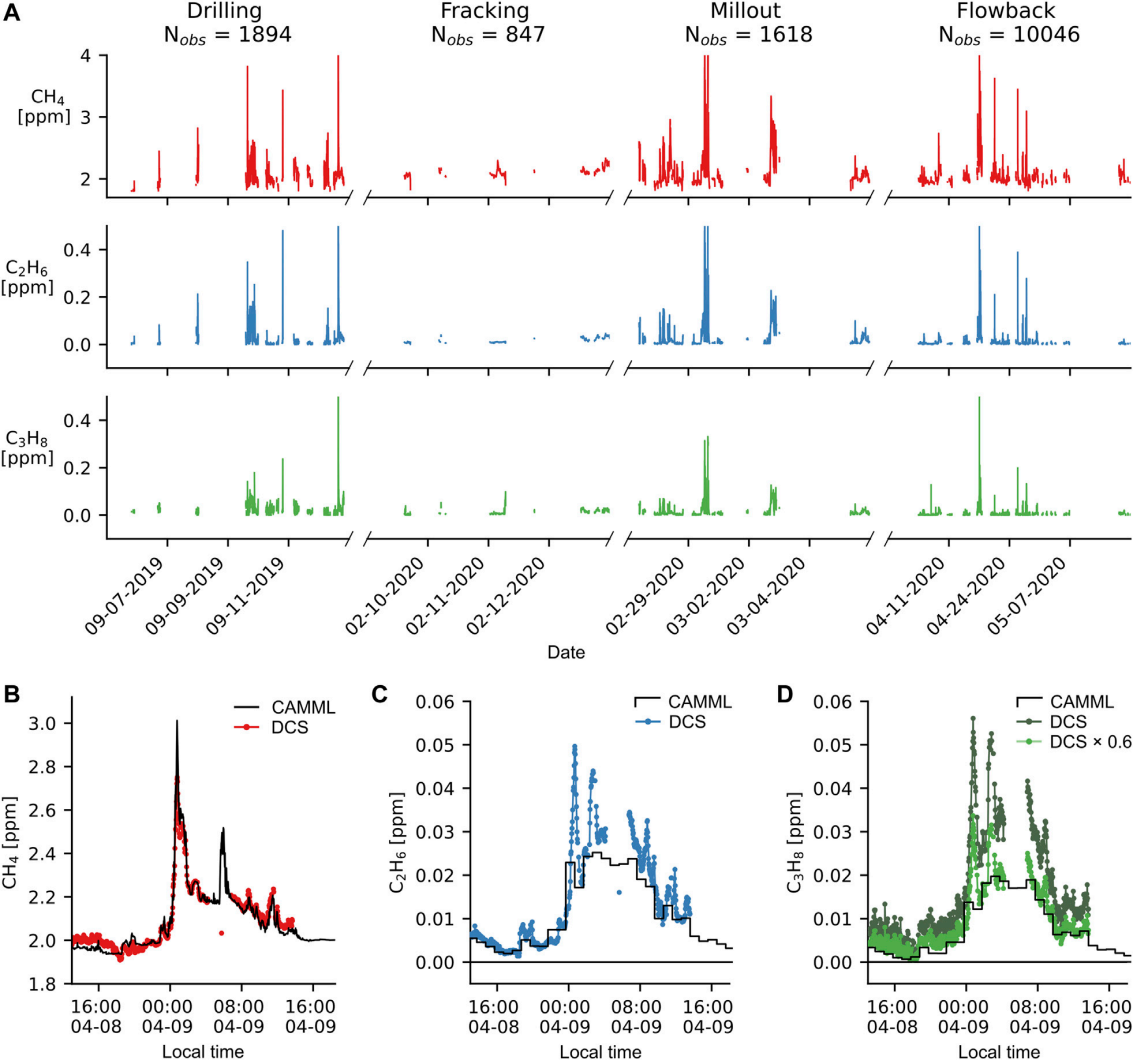
**(A)** Time series of methane (CH_4_), ethane (C_2_H_6_), and propane (C_3_H_8_) measured during different stages of well development, with the total number of 2-min observations during each phase listed in the sub-plot titles. Transient plumes of natural gas create large enhancements of methane, ethane, and propane mixing ratios. Less plume activity is observed during fracking compared to the drilling, millout, and flowback stages. **(B)** A representative methane time series demonstrates excellent agreement between the CAMML OA-ICOS and DCS measurements **(C)** Ethane and **(D)** propane data from the same period as **(B)** demonstrate the CAMML GC-FID and DCS capture the same hourly trends in the two VOCs, although the DCS data resolves the faster fluctuations in mixing ratios. Propane mixing ratios are initially 40% too large but match the CAMML data well after multiplying with a 0.6× correction factor.

The DCS system was deployed four times to collect data across multiple months as the well installation progressed through different phases. Time series of retrieved gas mixing ratios measured by the DCS system (filtered for data with reliable retrievals) are shown in Figure 3A. DCS and CAMML measurements occurred during four major phases of well pad development: drilling, fracking, millout, and flowback/production. Weather and temperature fluctuations (especially during the fall and winter of 2019-2020 before the van temperature control and insulation were improved) did result in some outages which prevented full monitoring of all phases. DCS retrieval accuracy is evaluated by comparing to the CAMML data during a time where both instruments were measuring the same air mass. As seen in Figure 3B, the DCS methane time series closely tracks the CAMML OA-ICOS methane data, indicating that the DCS retrieval independently obtains the same concentrations as the OA-ICOS and with similar precision. Ethane measured by the DCS (Figure 3C) follows the hourly trends in concentration measured by the CAMML GC-FID while also detecting many short-lived plume events which are smoothed out by the GC-FID sampling. Propane (Figure 3D) agrees less well with the hourly trend in the GC-FID data, appearing to be ∼40% too high. We attribute this difference to errors in the spectral fitting, where excess spectral intensity from heavier alkane C-H stretches gets attributed to the rather amorphous propane spectrum. All DCS propane data were multiplied by a factor of 0.6 to improve agreement with the CAMML. Precisions were estimated as the standard deviations of methane (σ^2^ = 3.5 ppb), ethane (σ^2^ = 0.2 ppb), and propane (σ^2^ = 1.6 ppb) across a 1-h time period with minimal mixing ratio variations.

### 3.2 O&G emissions during well development phases

**FIGURE 4:**
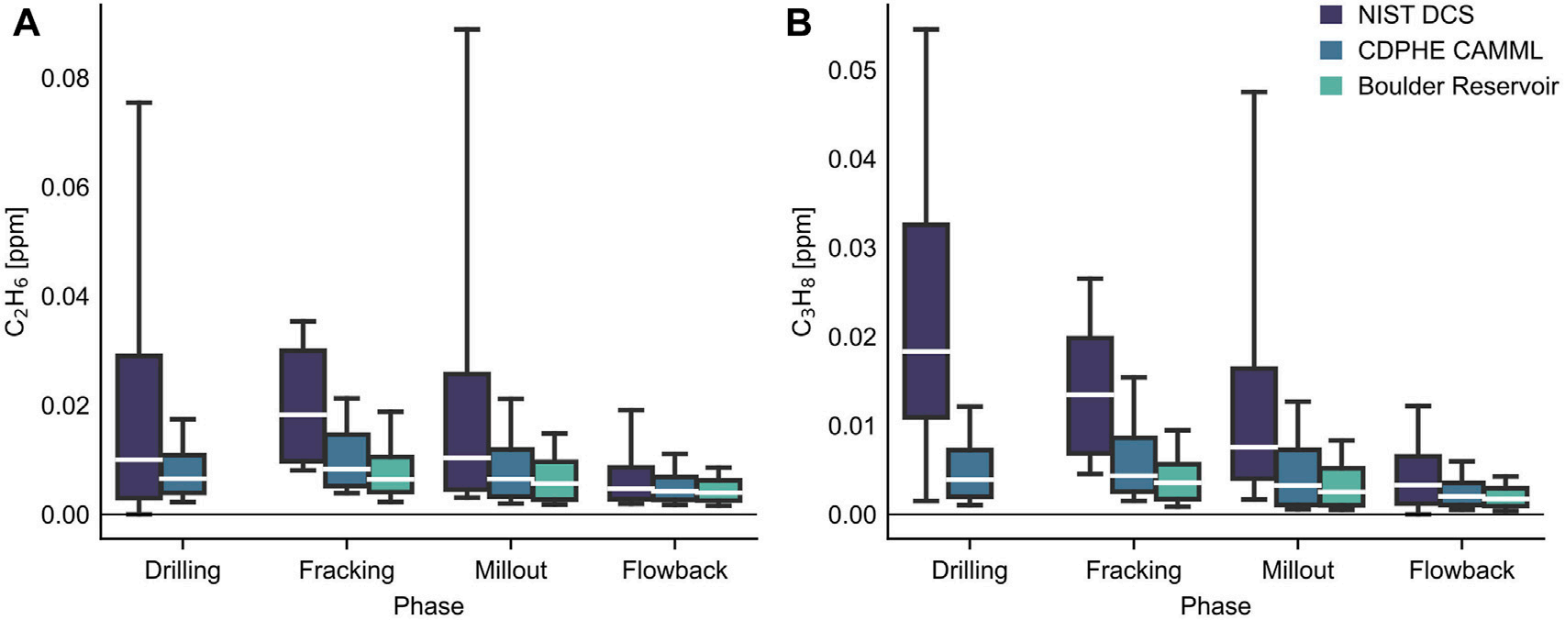
Box plots of ethane **(A)** and propane **(B)** mixing ratios for each phase of well development measured with the mid-IR DCS instrument (at 2-min resolution, in dark purple) are compared to the CDPHE CAMML instrument suite (at 45-min resolution, in blue), as well as the regional background values measured at an air quality site at Boulder Reservoir during the same time periods, in green. Ethane and propane mixing ratios are elevated relative to background until the flowback phase. Median values are shown as a white line, while the box edges are the first and third quartiles (Q1 and Q3) and the whiskers are set to the 10th and 90th percentiles. Outliers aren’t shown in these plots; this serves to highlight that the fracking phase, despite a lack of detected plumes, had median ethane and propane mixing ratios comparable to the drilling and millout phases. Ethane and propane data were not available from the Boulder Reservoir site during the drilling phase.

Thermogenic methane characteristic of oil and natural gas deposits is frequently rich in ethane and other alkanes. Therefore, comparing the DCS ethane and propane concentrations to a distant regional background can indicate the presence of nearby excess O&G emissions. Ethane and propane data from a long-term air quality monitoring site at the Boulder Reservoir (40.0700, −105.2202, located ∼18.5 km to the west of the well pad) spanning the measurement time periods was used for comparison (Boulder County Public Health, 2020). Summaries of ethane and propane enhancements observed during each phase are shown in Figures 4A, B. Both VOC mixing ratios observed with the DCS instrument at Broomfield were enhanced compared to the background Boulder Reservoir site during the drilling, fracking, and millout phases, consistent with past observations (Howarth et al., 2011). Flowback of the well coincided with a reduction of ethane and propane concentrations to near background levels. Large plumes of methane, ethane, and propane were less frequently observed during the fracking stage, although median mixing ratios remained comparable to the drilling and millout phases. This observation may suggest different emissions mechanisms are operative during the fracking phase; for example, large plumes may be more likely when the well bore is more frequently exposed, as it is during drilling and millout compared to fracking. The CDPHE CAMML methane sensor similarly observed many fewer plume events during the entire fracking period than during drilling and millout phases. The observations are also consistent with the lower emission rates observed by Hecobian et al. (2019) in the Denver-Julesburg basin during fracking compared to millout/flowback. Note drilling wasn’t measured during that study for the DJ basin.

Notably, the open-path instrument also consistently observed higher median ethane and propane mixing ratios than the CDPHE CAMML point sensor instrument. This is consistent with the observation that the DCS time series in Figures 3C, D resolved large plumes that the CAMML GC-FID doesn’t detect due to the different averaging techniques (2 min averaging over the open path with nearly 100% duty cycle vs. 45 min adsorbing onto a sample matrix with 75% duty cycle). Additional reasons for the higher median values in the DCS data could be due to the different measurement approaches (point vs. integrated path) and the active switching of the DCS beam between retros to more frequently measure downwind of the well pad. Also potentially contributing to the difference in the case of propane is some residual degree of fit uncertainty remaining after the CAMML-derived correction factor.

### 3.3 Plume identification

**FIGURE 5:**
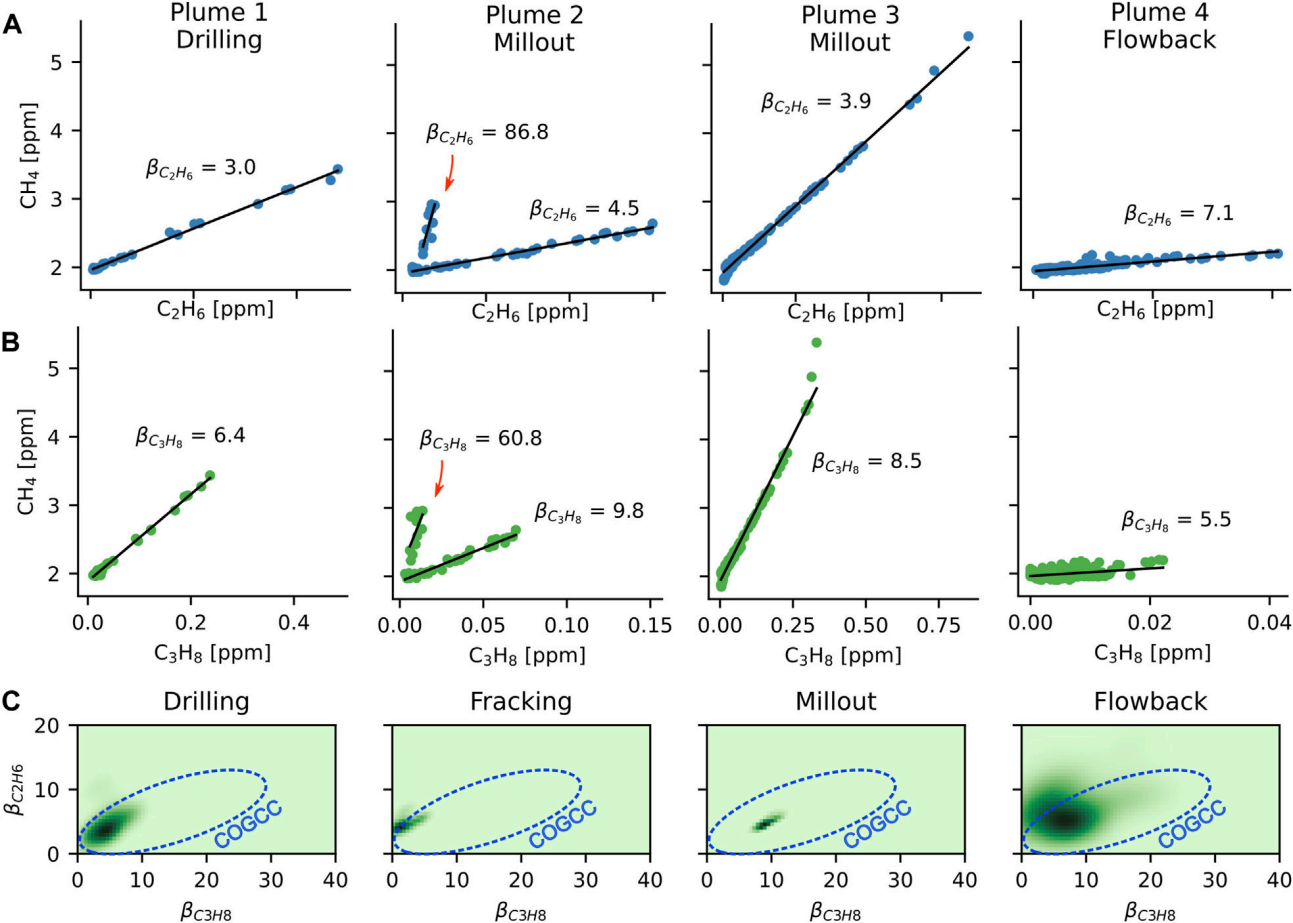
**(A)** Correlation plots of ethane and methane for several discrete O&G plumes are shown, along with the enhancement ratio derived for each plume. Note the clear separation of methane with small and large $\beta_{C2H6},\beta_{C3H8}$ coefficients in the Plume 2 data. **(B)** Same plume events are shown as in A, but showing correlations between propane and methane. Again, there are large variations in enhancement ratios. Note that the *x*-axis scale varies between panels in **(A, B)**, but is shared across ethane and propane for each plume. **(C)** Density estimates of the ethane and propane enhancement ratios calculated using the dynamic linear model for each phase of well development. Median COGCC results are shown by the oval contour lines. COGCC estimates are the same for all panels, reflecting the multi-year nature of the COGCC data.

Many potential methane sources exist around the measurement location, including not only well pad emissions but also methane from nearby landfills. Over short (few hour) time periods, individual methane plumes from the well site are expected to have strong, well defined correlations with ethane and propane (Yacovitch et al., 2014). Due to the high time resolution of the DCS system, it is possible to attribute individual methane plumes to the well site based upon this methane-VOC correlation. In Figures 5A, B, we highlight several characteristic methane plumes with large (thermogenic) ethane and propane mixing ratios. We can quantify the methane-VOC correlation by calculating a VOC enhancement ratio; these are determined by using a linear regression model to model the observed methane as a background term *β*_0_ and an enhancement ratio ( $\beta_{C2H6},\beta_{C3H8}$ ) scaled by the concentration of one of the VOCs (Kille et al., 2019). Note that there is a high degree of collinearity between ethane and propane, as both molecules are tracers for methane from oil and natural gas sources. Analyzing the ethane-methane and propane-methane correlations separately is necessary to retrieve meaningful regression coefficients.

$$CH_{4}=\beta_{0}+\beta_{C2H6}C_{2}H_{6}$$ (1)

$$CH_{4}=\beta_{0}+\beta_{C3H8}C_{3}H_{8}$$ (2)

This approach allows O&G plumes from the well site to be separated from other nearby methane sources. An example of this separation is shown in the plume 2 event, which was detected close in time with a second, VOC-depleted methane plume indicated by the red arrows in Figures 5A, B. While it is difficult to precisely identify the source of this plume, the northerly wind during this time period corresponds to side-by-side natural gas production and landfill facilities located ∼5.5 km to the north.

While methane/VOC enhancement ratios from different plume events can potentially provide information about the exact source of the emissions (Cardoso-Saldaña et al., 2019), it is useful to analyze the entire time series to understand how the βC2H6 and βC3H8 enhancement ratios may vary between different phases. A dynamic linear model (DLM) is used to extract the enhancement ratios of ethane and propane in the natural gas plumes (West and Harrison, 1997; Mead et al., 2023). For both ethane and propane, a DLM was applied to the time series data for each operational phase to generate a time-varying set of *β*_0_ and $\beta_{C2H6},\beta_{C3H8}$ parameters. Correlations between the $\beta_{C2H6}$ and $\beta_{C3H8}$ time series produced by the DLM for each phase of development are shown as density plots in Figure 5C. We can compare these enhancement ratios to data provided by the Colorado Oil and Gas Conservation Commission, which report the chemical composition of thousands of samples collected from wells throughout the DJ basin (Colorado Oil and Gas Conservation Commission, 2022). Estimates from the COGCC data are shown by the oval contour in Figure 5C. Enhancement ratios for ethane and propane are on the lower end of COGCC samples, and were also slightly lower than other measurements performed in the same region (Pétron et al., 2014). Notably, however, the COGCC samples and previous studies did not target the well development process. Our results indicate that emissions from well development are more alkane rich than those observed during standard well operations, which may indicate a diverse range of sources including not only well borehole emissions but also potentially emissions from vehicles and generators and compressors. Clearer documentation of the equipment and techniques employed at well sites during development, and how these practices produce large variability in the VOC/methane ratios will be critical for improving emissions attribution to different portions of the O&G extraction and processing pipeline.

### 3.4 DCS-AERMOD methane flux estimation

The complex topography around the well site as well as the high sound walls encompassing the site precludes reliable application of a simple Gaussian plume model or Lagrangian model to estimate the magnitude of emissions from the well site for this study. However, to illustrate the potential of estimating fluxes using the DCS instrument and knowledge of the measurement path, we use AERMOD (Cimorelli et al., 2005), modified to run at high time resolution, to estimate the emissions from one plume event. This plume event was identified for modeling because we have high confidence it originated from the well pad based upon the differential retro path measurements performed before and during the event. AERMOD was chosen because it can account for some terrain features and has a model for building downwash, which we use here to represent the sound wall around the site. We represent each measurement path by an array of point receptors, and then determine the path-averaged concentration enhancement predicted. A single point source was simulated at a release height of 10 m centered on the well pad (This height was chosen to match the sound wall height.). Taking the ratio of observed O&G enhancement to predicted enhancement produces a predicted emissions rate.

**FIGURE 6:**
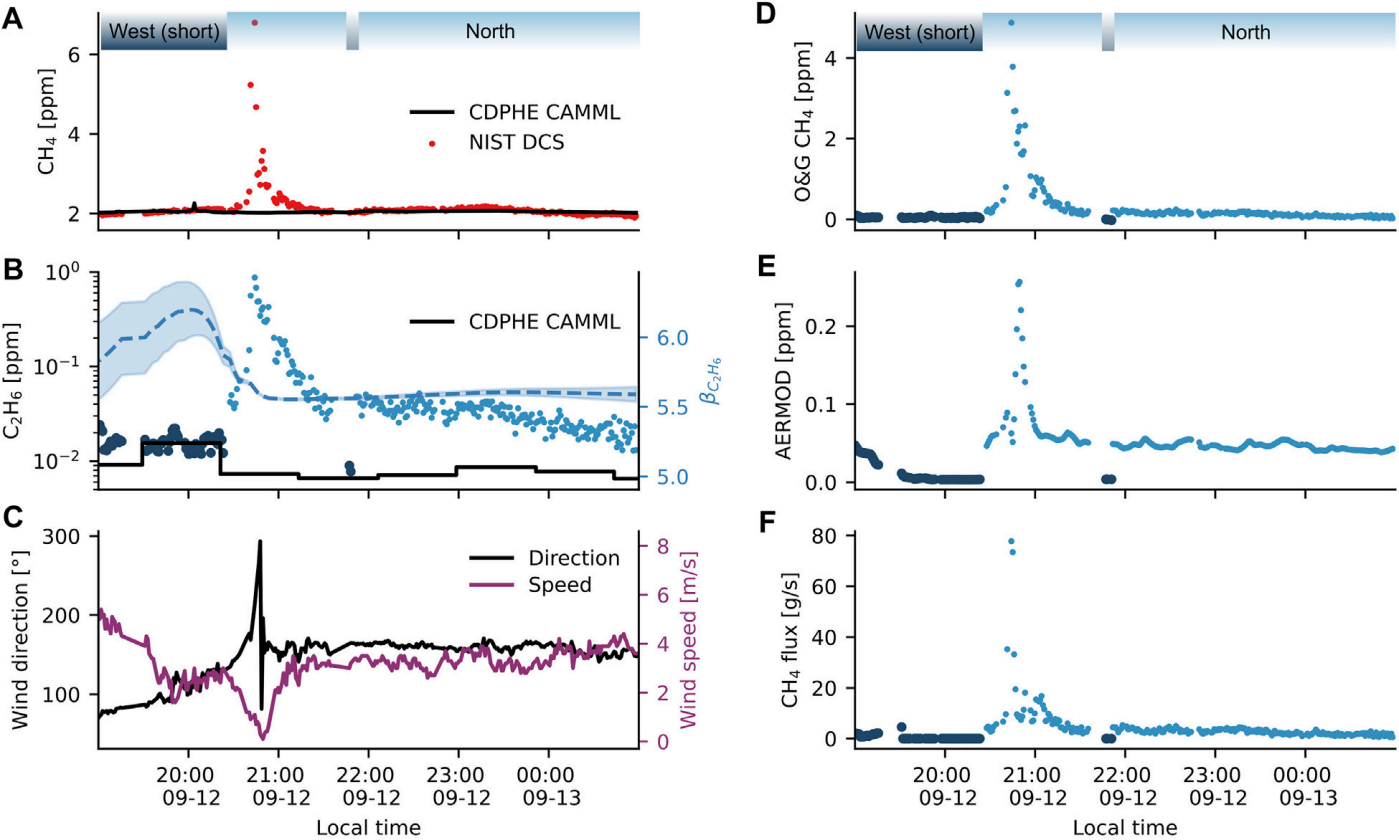
Emission estimate for plume from 12 September 2019. **(A)** Observed methane mixing ratios detected by the DCS and CAMML OA-ICOS instrument. The greater span and time resolution of the DCS technique captures the substantial plume which is undetected by the CAMML. Wind was constant for 2 hours at ∼152° up to and including the main plume event, which transported emissions to the north DCS path but not to the OA-ICOS inlet. **(B)** Ethane mixing ratios observed by the DCS and CAMML GC-FID instruments (left axis). The right axis shows how the DLM ethane enhancement ratio $\beta_{C2H6}$ (dashed blue line) and its uncertainty (shaded blue region) varies throughout the plume event. **(C)** Wind direction and speed during the plume event measured by the CAMML weather station. **(D)** Observed path-averaged methane plume measured at the short west and north retros. **(E)** AERMOD predicted path-averaged enhancement for the measurement paths shown in Figure 1. Note that the downwind path (north retro) is strongly enhanced relative to the crosswind path (short west retro). **(F)** Derived emissions rates in grams/second for the observed plume.

Figure 6A shows observations of methane for one plume event that was observed on the evening of 12 September 2019 during the drilling phase. The two measurement paths, indicated above the time series, help determine that the plume event arises from the well pad. From ∼7:00 p.m. to ∼8:30 p.m., measurements were made along the short west retro path; the wind direction during this period (Figure 6C) trended from ∼75°–130° and would have begun to transport any emissions from the well to the short west path. Despite the favorable wind direction, there was no observable enhancement on the short west path (although the wind direction does transport a small methane enhancement west to the CAMML OA-ICOS instrument at ∼8 p.m.). Shortly after changing the DCS path to the north path around 8:30 p.m., the wind direction shifts to ∼152° and the wind speed drops. The combination of direct wind transport from the well pad to the north path and the decreased wind speed leads to a large plume event around 9 p.m. on the north path.

AERMOD simulations of enhancements on each retro path assuming a 1 g per second emission from the well pad confirmed that enhancements were only predicted on the north path of the well pad (Figure 6E) and thus likely undetectable by the CAMML located west of the well pad. Using the dynamic linear model analysis of ethane (Figure 6B), the O&G component of the methane time series ( $CH_{4}^{O\&G}=\beta_{C2H6}C_{2}H_{6}$ ) was extracted from the methane time series (Figure 6D). Taking the ratio of the observed and predicted enhancements produced an estimated emissions rate which peaked at over 80 g CH_4_/s (Figure 6F). After 9:30 p.m., a steady emission rate of several g CH_4_/s is observed until around 12 a.m. While these values are consistent with fluxes observed at other drilling sites (Caulton et al., 2014), it is not possible to determine if this plume is due to an actual change in emissions or was a consequence of pooling and ventilation from the site.

This result shows the potential for open-path DCS to measure emissions at sites like this; however, we emphasize that the numbers are only given as estimates and have potentially large uncertainties. No plume models have been validated with open-path measurements at sites like the drilling site. Thus, more measurements with controlled releases as well as a comparison of different models (including potentially large-eddy simulations) would be necessary to test the validity of the measurements. Because of this, we don’t estimate emissions for other plume events. In future measurements, where the measurement geometry is less constrained by the surrounding urban environment, it would also be possible to move the beam paths further from the sounding walls and reduce our sensitivity to their effects.

## 4 Discussion

Continuous open-path measurements during drilling operations can provide valuable information for public health studies. We note that the largest plumes were observed at nighttime, when stagnant atmospheric conditions lead to pooling of emissions. While average daily VOC enhancements might be low, these pooling events can lead to very high enhancements, which is an important consideration for potential health and air quality impacts. Furthermore, these events are hard to model with atmospheric dispersion models, so *in situ* measurements are necessary to capture the frequency of such stagnation events. We observed many plume events with the DCS that were not resolved by the CAMML due to both the wind direction and the lower temporal resolution of the CAMML GC-FID. These events include several large events with methane concentrations exceeding 6 ppm such as that shown in Figure 6A This ability to capture more plumes and temporal dynamics highlights an important advantage of the open-path measurements for understanding the health and air quality impacts of oil and natural gas emission. For example, while we did not measure benzene, we can use the range of benzene/ethane ratios measured by the CAMML during other plumes to estimate that the benzene in the plume shown in Figure 6 likely peaked somewhere between 10 ppb and 50 ppb–highlighting the need for further measurements and more attention to optimal location of point and path sensors.

Since this was an initial test deployment, long-term measurements were hampered by instabilities with the laser system primarily due to large (>20°C) temperature fluctuations in the van adversely impacting the temperature sensitive DFG process. This was improved at the later stages of the deployment. In addition, recent work in our group has demonstrated substantially greater up-time with a similar mid-IR DCS system by minimizing thermal drifts and optical misalignment, as well as by incorporating a slow feedback to optimize spectral power during observations (Herman et al., 2023), which will enable continuous monitoring in the future.

Finally, more VOCs than reported here can potentially be measured in the future. HCHO can be retrieved from its absorption around 2,800 cm^−1^. Heavier alkanes have absorption features in this spectral region and could potentially be retrieved with different analysis methods. Methanol and formic acid have been observed in laboratory combustion using DCS in the same spectral region (Makowiecki et al., 2021). Aromatics such as benzene and toluene have absorption features near 3,100 cm^−1^ that could potentially be detected with improved DFG to produce more light in this region.

## Data Availability

The raw data supporting the conclusion of this article will be made available by the authors, without undue reservation.
